# Effectiveness of the Auditory Temporal Ordering and Resolution Tests to Detect Central Auditory Processing Disorder in Adults With Evidence of Brain Pathology: A Systematic Review and Meta-Analysis

**DOI:** 10.3389/fneur.2021.656117

**Published:** 2021-06-02

**Authors:** Sanathorn Chowsilpa, Doris-Eva Bamiou, Nehzat Koohi

**Affiliations:** ^1^The Ear Institute, University College London, London, United Kingdom; ^2^Otology Neurotology and Communication Disorder Unit, Department of Otolaryngology, Faculty of Medicine, Chiang Mai University, Chiang Mai, Thailand; ^3^Neuro-Otology Department, University College London Hospitals, London, United Kingdom; ^4^Biomedical Research Centre, National Institute for Health Research, London, United Kingdom; ^5^Department of Clinical and Movement Neurosciences, Institute of Neurology, University College London, London, United Kingdom

**Keywords:** auditory temporal processing, temporal ordering test, temporal resolution test, gaps-in-noise test, frequency pattern test, duration pattern test

## Abstract

**Background:** Auditory temporal processing tests are key clinical measures in order to diagnose central auditory processing disorder (CAPD). Although these tests have been used for decades, there is no up-to-date evidence to determine the effectiveness of detecting the abnormalities in central auditory processing in adults while the available national CAPD guidelines predominantly address CAPD in the pediatric population.

**Purpose:** To determine the efficacy of the auditory temporal ordering tests [duration pattern test (DPT) and frequency pattern test (FPT)], and a temporal resolution test [gaps-in-noise (GIN) test] for detecting the central auditory processing abnormalities in adults with documented brain pathology.

**Research Design:** Systematic reviews and meta-analyses.

**Study samples:** Four databases, including PubMed, Web of Science, Embase, and Scopus, were systematically searched. The publications in the English language that recruited adults (above 16 years old) with pathologic brain conditions and described the diagnostic tests for auditory temporal processing were selected for review.

**Data Collections and Analysis:** All data were systematically evaluated, extracted, categorized, and summarized in tables. The meta-analysis was done in order to determine the effectiveness of the DPT, FPT, and GIN tests.

**Results:** The results showed significantly poorer performance of DPT and FPT, compared between participants with confirmed brain disease and normal controls, at the mean differences of percent correct −21.93 (95% CI, −26.58 to −17.29) and −31.37 (95% CI, −40.55 to −22.19), respectively. Subjects with brain pathology also performed poorer in GIN test at the mean difference of 3.19 milliseconds (95% CI, 2.51 to 3.87).

**Conclusion:** The results from the meta-analysis provide evidence that DPT, FPT, and GIN clinical measures are effective in the diagnosis of CAPD in adults with neurological disorders. Poor performance on these tests is significantly related to the confirmed brain pathology. However, different units in results presentation and variety of testing strategies are limitations for this meta-analysis. The standard pattern of result reporting and international protocols test strategies should be developed in order to conduct better meta-analyses with a larger collection of suitable studies and less heterogeneity.

## Introduction

### Definition of Auditory Processing Disorder

Central auditory processing disorder (CAPD) is a clinical diagnosis that is characterized by normal (or near-normal) hearing thresholds and a variety of hearing symptoms, including difficulties understanding speech in noisy environments, discriminating speech, localizing sounds, auditory inattention, or memory difficulties, that arise due to abnormal auditory processing within the brain. Individuals with CAPD require multisensory cues to support their listening and may experience cognitive or language difficulties (1). These auditory difficulties are not specific to CAPD, as they may also present in other disorders, for example, autism spectrum disorder (ASD), attention deficit disorder, or cognitive disorders (1).

There is a variety of tests that assess the different auditory processing domains, including auditory discrimination, temporal processing, dichotic listening, low-redundancy speech recognition (monaural), and binaural interaction (2). Baseline audiological assessments such as standard pure-tone audiometry, speech-in-quiet audiometry, otoacoustic emissions, and electrophysiological measures are also important when assessing for CAPD in order to control for the presence of peripheral auditory impairment, and complement behavioral auditory processing tests.

### Overview of Central Auditory Processing and Tests

The central auditory nervous system (CANS) starts at the cochlear nucleus in the brainstem and extends up to the primary auditory cortex and association cortices (3). The peripheral sensory input is transferred ipsilaterally and contralaterally. This signal is processed in serial and parallel at various levels of the auditory pathway, resulting in the complex processing of auditory signals (3). The CAPD battery tests are not site-specific. For example, temporal ordering and dichotic listening tests could both detect abnormalities of bilateral auditory cortex and interhemispheric function, as well as abnormalities in the brainstem (3). Because of the complex structure of CANS with its overlapping multilevel neural networks that subserve different facets of auditory processing, a single CAPD test may not sufficiently diagnose CAPD (2, 3). Multiple tests are needed to evaluate the function of the CANS and determine the possible site of lesion in order to explain the patient-reported listening difficulties.

Although there are various types of tests for CANS assessment, this review focuses on the studies that evaluate the ability of auditory temporal processing tests that are commonly used in clinical applications for central auditory processing evaluation (3, 4).

### Auditory Temporal Processing Assessment

Auditory temporal processing is important for the detection and discrimination of syllable, phoneme, and stress patterns and phonological awareness. Temporal processing of rhythm, meter, and tempo also supports musical perception (5). There are four subprocesses of auditory temporal processing: (1) temporal ordering or sequencing, (2) temporal resolution or discrimination, (3) temporal integration, and (4) temporal masking. The first two subprocesses are more established in clinics in order to evaluate the central auditory processing function of patients, since there are no commercially available tests of temporal masking and temporal integration (6). Thus, this review focuses on studies that evaluate auditory temporal ordering and temporal resolution processing.

#### Temporal Ordering Tests

The temporal ordering tests refer to the processing of the presence of different auditory stimuli in the pattern of occurrence in defined time (6). The frequently used clinical tests for temporal ordering evaluation are duration pattern test (DPT) and frequency pattern test (FPT) (2, 7, 8). Temporal ordering tests provide the information on bilateral hemispheres function, integration *via* the corpus callosum, and association to cognitive and perceptual processes (6). DPT and FPT have good sensitivity, specificity, and test–retest reliability for patients with cerebral lesions (7–9). However, FPT was less sensitive to the brainstem lesions compared to the cerebral lesion at 45% and 83%, respectively (8). In contrast, DPT is more likely to be abnormal in brainstem lesions (9). Although DPT and FPT are both temporal ordering tests, there is no correlation between the two (10), so they cannot be used alternatively.

#### Temporal Resolution Tests

Temporal resolution tests assess the shortest duration that subjects can distinguish between two auditory stimuli (6). The gaps-in-noise (GIN) test became popular because it can be applied in subjects with cognitive problems or peripheral hearing loss at a specific frequency (2, 11). Musiek et al. (12) showed a significantly poorer GIN performance in a neurological group with confirmed CANS involvement compared to normal controls. They proposed that the GIN test can be reliably used to detect abnormalities in central auditory processing particularly for lesions at the auditory cortex level. They also reported good test–retest reliability. However, there are some limitations of GIN test in that it is time-consuming and may not be sensitive to detect lesions at the brainstem (6, 12).

### Rationale and Aim of the Study

The majority of published CAPD guidelines discuss auditory processing assessments in children. Little can be found on CAPD in adults in these guidelines. Most current papers report the use of CAPD tests to evaluate adults with documented underlying diseases, such as stroke, that may affect central auditory processing. There is no uniformly accepted gold standard diagnostic battery or diagnostic criteria for CAPD. In the absence of this, it has been argued that auditory processing tests should be evaluated against documented CANS lesions to establish their sensitivity and specificity (2, 12–14).

The aim of this review paper is to determine the efficacy of temporal ordering tests (DPT and FPT) and a temporal resolution test (GIN test) and provide scientific evidence of their value in detecting central auditory processing abnormalities in individuals with documented adult-onset brain disorders who have well-established and/or documented brain pathology.

## Materials and Methods

### Database Searching

The protocols of Preferred Reporting Items for Systematic reviews and Meta-Analyses (PRISMA) guideline (15) and the Cochrane guideline for the intervention and the diagnostic test accuracy (16, 17) were followed. Four databases (PubMed, Web of Science, Embase, and Scopus) were systematically searched. These four medical databases provide comprehensive search result using keywords that automatically match the MeSH terms. The inclusion criteria were (1) studies describing the tests for auditory temporal ordering or temporal resolution, (2) studies with only adult participants aged above 16 years old with documented brain diseases, and (3) publication in the English language. The studies that recruited children participants or published in non-English language were excluded from this review. The studies that evaluated the auditory processing using only electrophysiological or imaging investigations without the behavioral auditory processing tests were excluded. Also, studies that conducted the tests in adult participants with cognitive, psychological/psychiatric, or developmental (early-onset) disorders were excluded.

The publications in the past 20 years were searched on the four databases. The database searching was completed on September 15, 2020, by SC and NK using the search terms “duration pattern test”, “frequency pattern test”, “GIN”, “gaps in noise”, “temporal resolution”, “temporal discrimination”, “temporal ordering”, and “temporal sequencing” combined with “auditory process^*^.” After the duplicates were removed, in order to avoid bias, the eligibility of papers was independently reviewed by at least two authors at each key step, including abstract screening, full-text reading, and data extraction. The results of assessments were then compared and determined for agreement in order to include the suitable studies for the systematic review and meta-analysis. If the consensus could not be reached by the two researchers, the studies were further evaluated and judged by a third reviewer. The risk of bias of the selected studies was evaluated following the QUADAS-2 (quality assessment of diagnostic accuracy studies) (18) and the Newcastle–Ottawa Scale (NOS; for case control studies) (19). The PICO format (20), P—patient, problem, or population; I—intervention; C—comparison, control or comparator; O—outcome, was applied to compare the auditory temporal processing performance between the normal individuals and individuals with underlying brain disease as follows:

P: Adult participants without cognitive problems who underwent auditory temporal processing tests

I: Auditory temporal ordering tests or auditory temporal resolution tests

C: Comparison of results from each test between the normal participants and participants with underlying neurological brain conditions

O: Auditory temporal processing function.

### Data Analysis and Meta-Analysis

The studies that met the eligibility criteria were included in this review. All studies were categorized by the subtypes of the auditory temporal processing tests and subsequently classified by underlying conditions of participants. Participants with neurologic adult-onset brain conditions were diagnosed on the basis of neuroimaging and/or electroencephalography (EEG) investigations: computerized tomography (CT) scan, magnetic resonance imaging (MRI), other neuroimaging, or EEG, and/or a formal diagnosis of neurological brain disorder made by a medical specialist according to accepted diagnostic criteria. All data were systematically evaluated, extracted, categorized, and summarized in tables. To assess the effectiveness of the tests, only studies that recruited participants with confirmed brain disorders with control comparison were considered for further meta-analysis by using the evidence of documented brain lesions as a gold standard.

To perform the meta-analysis, the studies that reported participants with documented structural or functional brain lesions were considered for eligibility, depending on the presence of comparison between the disease and control groups and the similarity of the unit reported in the result of each study. The Review Manager (RevMan) program version 5.4.1, suggested by the Cochrane Collaboration (21), was applied for meta-analysis. The random effect model with 95% confidence interval (CI) difference was applied in order to compare the mean difference between the normal control groups and the documented organic brain groups. The results from the meta-analysis were interpreted in terms of the mean difference, 95% CI, and heterogeneity, determined by *I*^2^ statistic.

## Results

**Figure 1:**
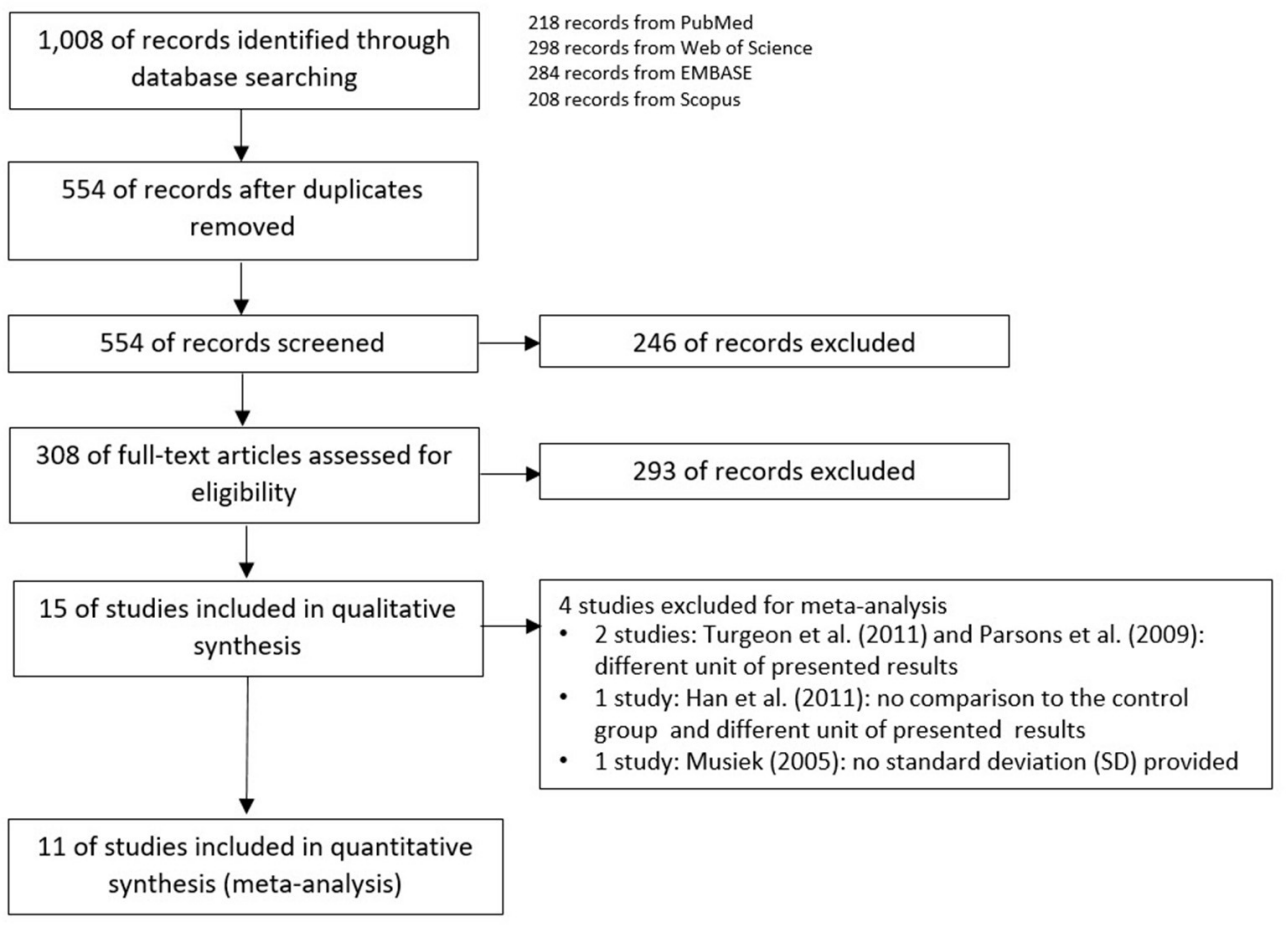
Preferred reporting items for systematic reviews and meta-analyses (PRISMA) flow diagram.

The schemes of database searching and the results according to the PRISMA guideline are shown in Figure 1.

**Figure 2:**
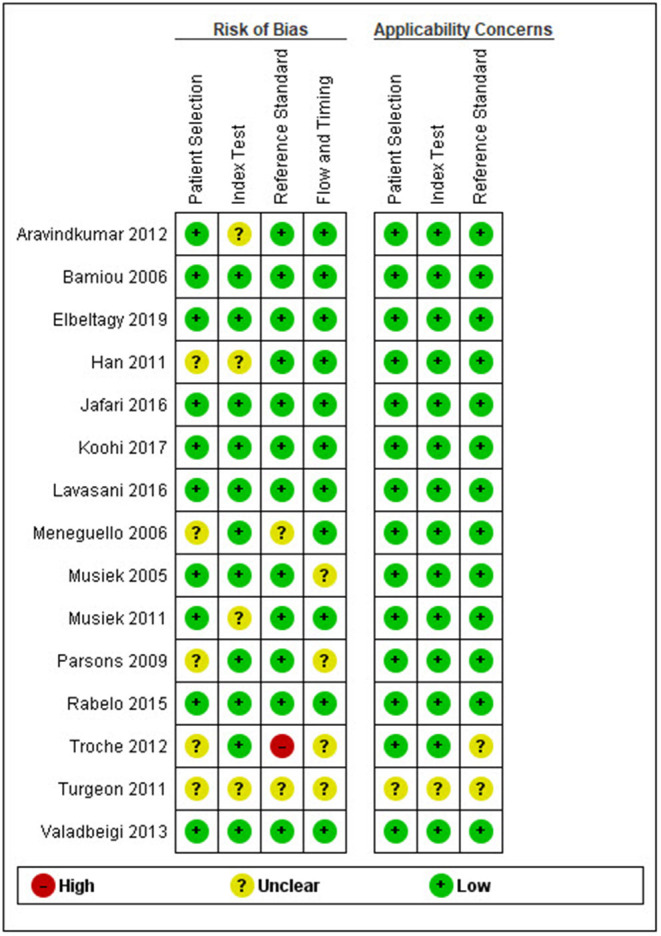
Risk of bias and applicability concern summary following the QUADAS-2 (quality assessment of diagnostic accuracy studies) criteria.

After duplicates and ineligible study exclusion and the full-text assessment, 15 studies were recruited in this review. The risk of bias evaluation results, using QUADAS-2 and NOS, were shown in Figure 2 and Table 1, respectively. Only 11 studies were included in the meta-analysis. Four studies were excluded due to the reasons shown in Figure 1. These studies were classified by the CAPD test categories and underlying conditions and summarized in Tables 2–4.

**Table 1:** Risk of bias evaluation results using the NOS for case control studies.

**Study ID**	**Selection**				**Comparability**	**Exposure**		**Total (8 ⋆)**
	**Is the case definition adequate? (⋆)**	**Representativeness of the cases (⋆)**	**Selection of controls (⋆)**	**Definition of controls (⋆)**	**(⋆⋆)**	**Ascertainment of exposure (⋆)**	**Same method of ascertainment for cases and controls (⋆)**	
Aravindkumar et al. (22)	⋆	⋆	⋆	⋆	⋆-	⋆	–	6
Bamiou et al. (23)	⋆	⋆	⋆	⋆	⋆⋆	⋆	–	7
Elbeltagy et al. (24)	–	⋆	⋆	⋆	⋆⋆	⋆	–	6
Jafari et al. (25)	⋆	⋆	⋆	⋆	⋆⋆	⋆	–	7
Koohi et al. (26)	⋆	⋆	⋆	⋆	⋆⋆	⋆	–	7
Lavasani et al. (27)	⋆	⋆	⋆	⋆	⋆⋆	⋆	–	7
Meneguello et al. (28)	–	⋆	⋆	⋆	⋆⋆	⋆	–	6
Musiek et al. (12)	⋆	⋆	⋆	⋆	⋆-	⋆	–	6
Musiek et al. (13)	⋆	⋆	⋆	⋆	⋆⋆	⋆	–	7
Parson et al. (29)	⋆	⋆	⋆	⋆	⋆-	⋆	–	6
Rabelo et al. (30)	⋆	⋆	⋆	⋆	⋆-	⋆	–	6
Troche et al. (31)	⋆	⋆	⋆	⋆	⋆-	⋆	–	6
Turgeon et al., (32)	⋆	⋆	⋆	⋆	⋆-	⋆	–	6
Valadbeigi et al. (33)	⋆	⋆	⋆	⋆	⋆⋆	⋆	–	7

*NOS, Newcastle–Ottawa Scale*.

^*^The study by Han et al. (34) was not included in NOS evaluation because there was no control in the study.

**Table 2:** Tests for auditory temporal ordering or sequencing: DPT in participants with documented organic brain diseases.

**Study**	**Comparison between disease and control group**	**Underlying conditions of participants**	**Group of participants in study**			**Analysis by RevMan**
			**Group 1 (control group)**	**Group 2**	**Group 3**	
**Bamiou et al**. **(****23****)**	Yes Total *n* = 16	Participants in the disease group had insular stroke, confirmed by MRI.	**Age and hearing matched controls** *n* = 8 Age = N/A M:F = N/A	**Insular stroke participants** *n* = 8 Age = 63 (range 36–79) M:F = 5:3	—	Yes
			**Results presented in percent correct (Mean** ***±*** **SD) without statistical evaluation**			
			RE = 89 ± 11 LE = 94 ± 9	RE = 43 ± 12 LE = 41 ± 9	—	
**Han et al**. **(****34****)**	No Total *n* = 28	Normal-hearing participants with documented TLE, diagnosed by clinical history and several investigations. They were also treated with AEDs.	—	**TLE participants** *n* = 28 Age = 38.3 M:F = 20:8 Average duration of having epilepsy = 20.0 ± 11.4 years	—	No (Due to no comparison to the control group and different unit of presented results)
			**Results presented in overall percentage of patients with abnormal score**			
			—	RTLE = 53.3% LTLE = 60% Bilateral TLE = 66.7%	—	
**Jafari et al**. **(****25****)**	Yes Total *n* = 70	Participants in the disease group had history of stroke, confirmed by MRI.	**Normal controls** *n* = 25 Age = 50.52 ± 9.65 M:F = 18:7	**Poststroke with RBD (right-brain damage)** *n* = 25 Age = 51.68 ± 10.18 M:F = 19:6 72% thrombotic stroke, 28% embolic stroke	**Poststroke with LBD (left-brain damage)** *n* = 20 Age = 52.91 ± 9.74 M:F = 14:6 64% thrombotic stroke, 36% embolic stroke	Yes
			**Results were presented in percent correct (Mean** **±** **SD)**			
			RE= 90.71 ± 7.16 LE = 91.81 ± 7.56	RE = 79.80 ± 9.35 LE = 73.04 ± 7.86 (significant difference from controls, *p* < 0.001)	RE = 62.49 ± 9.54 LE = 78.38 ± 9.05 (significant difference from controls, *p* < 0.001)	
				**Combined data of RBD and LBD groups**		
				RE = 72.1 ± 9.33 LE = 75.41 ± 8.31	
**Lavasani et al**. **(****27****)**	Yes Total *n* = 43	Participants in disease groups were diagnosed TLE with normal hearing threshold.	**Age-matched healthy controls** *n* = 18 Age = 29.4 M:F = N/A	**Right temporal lobe epilepsy (RTLE)** *n* = 11 Age = 31.1 M:F = N/A	**Left temporal lobe epilepsy (LTLE)** *n* = 14 Age = 31.1 M:F = N/A	Yes
			**Results were presented in percent correct (Mean** ***±*** **SD). Authors reported significant worse performance in LTLE groups, but there was no significant difference between control and RTLE groups**.			
			RE = 94.99 ± 4.9 LE = 92.6 ± 5.5	RE = 93.6 ± 6.04 LE = 93.6 ± 6.2	RE = 63.08 ± 2.2 LE = 63.50 ± 2.34	
				**Combined data of RTLE & LTLE groups**		
				RE = 76.51 ± 4.22 LE = 76.74 ± 4.36	
**Meneguello et al**. **(****28****)**	Yes Total *n* = 18	All participants had normal hearing. Participants in the disease group were confirmed their diagnosis with TLE and prescribed AED.	**Normal controls** *n* = 10 Age = 29 M:F = 4:6	**TLE participants** *n* = 8 Age = 36.38 M:F = 4:4	—	Yes
			**Results presented in percent correct (Mean** **±** **SD) without statistical evaluation**			
			RE = 85.2 ± 17.33 LE = 87.5 ± 15.78	RE = 53.25 ± 26.87 LE = 56.62 ± 28.16	—	
**Troche et al**. **(****31****)**	Yes Total *n* = 27	Participants in disease groups had mild to moderate Parkinson's disease [defined by Hoehn and Yahr (35), Stage II–III classification].	**Age-matched controls** *n* = 12 Age = 70.3 ± 5.9 M:F = 8:7	**Parkinson's disease** *n* = 12 Age = 68.3 ± 8.7 M:F = 7:5	—	Yes
			**Results presented in percent correct (Mean** **±** **SD)**. **Authors concluded no significant difference between groups, F**_**(1, 25)**_, ***p*** **>** **0.05**			
			DurS = 92.50 ± 10.35 DurL = 100.00 ± 0	DurS = 86.46 ± 24.69 DurL = 95.83 ± 14.43	—	
**Turgeon et al**. **(****32****)**	Yes Total *n* = 16	Participants in disease groups had history of sport-related concussions, identified by the criteria from the American Academy of Neurology (Practice parameter 2000) and all athletes completed a Post-Concussive Symptom Checklist (Aubry et al., (36))	**Normal controls** *n* = 8 Age = 22.1 ± 1.6 M:F = N/A	**Concussed group** *n* = 8 Age = 23.5 ± 3.38 M:F = N/A	—	No (Due to different units of presented results)
			**Results presented in percentage of errors in individual participants without statistical evaluation performed**			
			N/A	Percent errors from 2 concussed subjects were 2 SD above those of the non-concussed group (subject 6: RE = 43% and LE = 17%; subject 8: RE = 13%)	—	
**Valadbeigi et al**. **(****33****)**	Yes Total *n* = 52	Participants in disease groups were diagnosed relapsing-remitting MS, by neurologists and MRI, with normal hearing threshold.	**Matched normal controls** *n* = 26 Age = 27.7 ± 5.2 M:F = N/A	**MS** *n* = 26 Age = 28.9 ± 4.1 M:F = N/A	—	Yes
			**Results were presented in percent correct (Mean** ***±*** **SD). Authors reported significant difference performance between groups**.			
			RE = 85.6 ± 6.5 LE = 86.4 ± 6.1	RE = 64.3 ± 6.9 LE = 67.6 ± 5.6	—	

*DPT, duration pattern test; M:F, male:female; AEDs, antiepileptic drugs; MS, multiple sclerosis; TLE, temporal lobe epilepsy; RE, right ear; LE, left ear; DurS, duration with small perceptual distance; DurL, duration with large perceptual distance; RTLE, right temporal lobe epilepsy; LTLE, left temporal lobe epilepsy. Age was presented in years: mean ± SD or mean (range)*.

**Table 3:** Tests for auditory temporal processing: FPT in participants with documented organic brain diseases.

**Study**	**Comparison between disease and control group**	**Underlying conditions of participants**	**Group of participants in study**		**Analysis by RevMan**
			**Group 1 (Control group)**	**Group 2**	
**Bamiou et al**. **(****23****)**	Yes Total *n* = 16	Participants in the disease group had insular stroke confirmed by MRI.	**Age and hearing matched controls** *n* = 8 Age = N/A M:F = N/A	**Insular stroke participants** *n* = 8 Age = 63 (range 36–79) M:F = 5:3	Yes
			**Results presented in percent correct (Mean** ***±*** **SD) without statistical evaluation**.		
			RE = 97 ± 6 LE = 96 ± 6	RE = 41 ± 30 LE = 48 ± 30	
**Elbeltagy et al**. **(****24****)**	Yes Total *n* = 40	Participants in the disease group were diagnosed MS	**Matched healthy controls** *n* = 20 Age = 37.3 ± 4.2 M:F = 12:8	**MS** *n* = 20 Age = 37.6 ± 5 M:F = 17:3	Yes
			**Results were presented in percent correct (Mean** ***±*** **SD). Authors reported significant difference between groups**.		
			RE = 85.8 ± 4.5 LE = 84.2 ± 4.7	RE = 70.3 ± 3.5 LE = 71.3 ± 4.1	
**Han et al**. **(****34****)**	No Total *n* = 28	Normal-hearing participants with documented TLE, diagnosed by clinical history and several investigations. They were also treated with AEDs.		**TLE participants** *n* = 28 Age = 38.3 M:F = 20:8 Average duration of having epilepsy = 20.0 ± 11.4 years	No (Due to no comparison to the control group.)
			**Results presented in the overall percentage of patients with abnormal scores**.		
			–	RTLE = 80% LTLE = 80% Bilateral TLE = 66.7%	
**Musiek et al**. **(****13****)**	Yes Total *n* = 49	Participants in the disease group had history of stroke, confirmed by MRI and CT	**Normal controls** *n* = 29 Age = 27.0 ± 10.5 M:F = 7:22 Normal hearing	**Stroke** *n* = 20 Age = 28.7 ± 12.2 M:F = 11:9 Site of lesion • 12 participants with right hemisphere lesions • 4 participants with left hemisphere lesions • 4 participants with bilateral lesions.	Yes
			**Results were presented in percent correct (Mean** ***±*** **SD). Authors claimed the significant difference between two groups without provided statistical data**.		
			RE = 94.06 ± 9.74 LE = 94.46 ± 7.95	RE = 43.35 ± 29.62 LE = 36.15 ± 29.51	
**Parsons et al**. **(****29****)**	Yes Total *n* = 30	Participants in the disease group were diagnosed with cerebellar degeneration with high functioning	**Normal controls** *n* = 15	**Cerebellar patient** *n* = 15	No (Due to different unit of presented result.)
			**Results were presented in mean pitch discrimination threshold. Authors reported 5.5 times difference between two groups (*****t*** **=** **4.34**, ***p*** **<** **0.0001)**		
			3.8 Hz (SD = 1.6)	20.9 Hz (SD = N/A)	
**Troche et al**. **(****31****)**	Yes Total *n* = 27	Participants in disease groups had mild to moderate Parkinson's disease, defined by Hoehn and Yahr (35) Stage II–III classification.	**Normal controls** *n* = 12 Age = 70.3 ± 5.9 M:F = 8:7	**Parkinson's disease** *n* = 12 Age = 68.3 ± 8.7 M:F = 7:5	Yes
			**Results were presented in percent correct (Mean** ***±*** **SD). Authors reported significant difference between groups [F**_****(1, 25)****_ **=** **16.75**, ***p*** **<** **0.05]**		
			DurS = 98.89 ± 4.3 DurL = 99.17 ± 3.28	DurS = 68.25 ± 36.65 DurL = 93.75 ± 18.07	
**Turgeon et al**. **(****32****)**	Yes Total *n* = 16	Participants in disease groups had history of sport-related concussions, identified by the criteria from the American Academy of Neurology (Practice parameter 2000) and all athletes completed a Post-Concussive Symptom Checklist (Aubry et al., (36))	**Normal controls** *n* = 8 Age = 22.1 ± 1.6	**Concussed group** *n* = 8 Age = 23.5 ± 3.38	No (Due to the difference of units in the presented result.)
			**Results presented in the percentage of errors of individual participants, without statistical evaluation**.		
			N/A	Percent of errors from 3 concussed subjects were normal values (subject 3: RE = 30%; subject 5: LE = 54%; subject 6: RE = 33% and LE = 40%).	

*FPT, frequency pattern test; M:F, male:female; AEDs, antiepileptic drugs; TLE, temporal lobe epilepsy; RE, right ear; LE, left ear; DurS, duration with small perceptual distance; DurL, duration with large perceptual distance; RTLE, right temporal lobe epilepsy; LTLE, left temporal lobe epilepsy. Age was presented in years: mean ± SD or mean (range)*.

**Table 4:** Tests for auditory temporal resolution or discrimination: GIN test in participants with documented organic brain disease.

**Study**	**Comparison between disease and control group**	**Underlying conditions of participants**	**Group of participants in study**			**Analysis by RevMan**
			**Group 1 (Control group)**	**Group 2**	**Group 3**	
**Aravindkumar et al**. **(****22****)**	Yes Total *n* = 76	All participants had normal hearing. Participants in disease group were diagnosed with refractory complex partial seizures and MTS.	**Normal healthy controls** *n* = 50 Age = 26.3 ± 5.17 M:F = 28:22	**Right MTS** *n* = 13 Age = 31.0 ± 7.67 M:F = 8:5	**Left MTS** *n* = 13 Age = 25.76 ± 8.26 M:F = 9:4	Yes
			**Results were presented in msec of GIN threshold (Mean** **±** **SD). Authors reported significant differences between groups**.			
			RE = 5.22 ± 1.11 LE = 5.06 ± 1.00	RE = 8.15 ± 2.34 LE = 7.85 ± 3.00	RE = 9.54 ± 3.67 LE = 10.15 ± 4.06	
				**Combined data of RTLE and LTLE groups**		
				RE = 8.85 ± 3.02 LE = 9.00 ± 3.50	
**Bamiou et al**. **(****23****)**	Yes Total *n* =16	Participants in disease group had insular stroke, confirmed by MRI	**Age and hearing matched controls** *n* = 8 Age = N/A M:F = N/A	**Insular stroke participants (RBD)** *n* = 5 Age = N/A M:F = N/A	**Insular stroke participants (LBD)** *n* = 3 Age = N/A M:F = N/A	Yes
			**GIN threshold results presented in msec (Mean** ***±*** **SD), without statistical evaluation**			
			RE = 4 ± 1 LE = 5 ± 1	RE = 8 ± 2 LE = 9 ± 1	RE = 11 ± 3 LE = 6 ± 2	
				**Combined data of RBD & LBD groups**		
				RE = 9.125 ± 2.20 LE = 7.875 ± 1.31	
**Elbeltagy et al**. **(****24****)**	Yes Total *n* = 40	Participants in the disease group were diagnosed MS	**Matched healthy controls** *n* = 20 Age = 37.3 ± 4.2 M:F = 12:8	**MS** *n* = 20 Age = 37.6 ± 5 M:F = 17:3	—	Yes
			**Results (GIN threshold) presented in msec (Mean** ***±*** **SD). Authors reported significant differences between groups**.			
			RE = 4.4 ± 0.5 LE = 4.7 ± 0.7	RE = 9.1 ± 1.0 LE = 9.8 ± 1.6	—	
**Jafari et al**. **(****25****)**	Yes Total *n* = 70	Participants in the disease group had history of stroke, confirmed by MRI.	**Normal controls** *n* = 25 Age = 50.52 ± 9.65 M:F = 18:7	**Post-stroke with RBD** *n* = 25 Age = 51.68 ± 10.18 M:F = 19:6 72% thrombotic stroke, 28% embolic stroke	**Post-stroke with LBD** *n* = 20 Age = 52.91 ± 9.74 M:F = 14:6 64% thrombotic stroke, 36% embolic stroke	Yes
			**Results (GIN threshold) presented in msec (Mean** ***±*** **SD)**			
			RE = 6.40 ± 1.84 LE = 6.52 ± 1.50	RE = 8.32 ± 3.21 LE = 9.56 ± 2.34	RE = 9.50 ± 2.39 LE = 8.15 ± 3.01	
				**Combined data of RBD and LBD groups**		
				RE = 8.84 ± 2.84 LE = 8.93 ± 2.63	
**Koohi et al**. **(****26****)**	Yes Total *n* = 82	Participants in disease groups had a history of stroke, confirmed by MRI. Their auditory functions were assessed at 3–12 months after the onset of stroke.	**Normal controls (from raw data)** *n* = 32 Age = 47.72 ± 13.06 M:F = 9:23	**Stroke (from raw data)** *n* = 43 Age = 58.07 ± 14.32 M:F = 34:9		Yes
			**Results were published in number of subjects with normal and abnormal results. However, the authors provided the raw data in order to recruit to the meta-analysis**.			
			RE = 5.94 ± 0.98 LE = 5.91 ± 0.73	RE = 7.93 ± 1.91 LE = 8.26 ± 2.71		
**Lavasani et al**. **(****27****)**	Yes Total *n* = 43	Participants in disease groups were diagnosed with TLE with normal hearing threshold.	**Age-matched healthy controls** *n* = 18 Age = 29.4 M:F = N/A	**RTLE** *n* = 11 Age = 31.1 M:F = N/A	**LTLE** *n* = 14 Age = 31.1 M:F = N/A	Yes
			**Results were presented in msec of GIN threshold (Mean** **±** **SD). Authors reported significantly worse GIN threshold in the TLE groups**.			
			RE = 4.77 ± 0.54 LE = 5.1 ± 0.83	RE = 7.09 ± 2.2 LE = 7.18 ± 2.3	RE = 6.64 ± 2.9 LE = 7.20 ± 2.6	
				**Combined data of RTLE and LTLE groups**		
				RE = 6.84 ± 2.56 LE = 7.19 ± 2.42	
**Musiek et al**. **(****12****)**	Yes Total *n* = 46	All participants had normal hearing. Participants in the disease group had confirmed neurological involvement to central auditory processing.	**Normal controls** *n* = 50 Age = 24.6 (range 13–46) M:F = 14:36	**Neurological participants** *n* = 18 Age = 46.4 (range 20–65) M:F = 14:4	—	No [Due to no standard deviation (SD) provided]
			**GIN threshold results presented in msec [Mean (range)]**			
			RE = 4.9 (4–8) LE = 4.8 (3–8)	RE = 8.5 (5–20) LE = 7.7 (5–15)	—	
**Rabelo et al**. **(****30****)**	Yes Total *n* = 46	All participants had normal hearing. Participants in disease group were diagnosed mesial temporal sclerosis.	**Normal controls** *n* = 30 Age = 24.9 ± 3.3	**Mesial temporal sclerosis** *n* = 16 Age = 38.9 ± 9.3	—	Yes
			**Results presented in msec of GIN threshold (Mean** **±** **SD)**			
			RE = 4.7 ± 1.0 LE = 4.6 ± 1.0	RE =7.4 ± 2.9 LE = 8.1 ± 1.7	—	
**Turgeon et al**. **(****32****)**	Yes Total *n* = 16	Participants in disease groups had a history of sport-related concussions, identified by using the criteria from the American Academy of Neurology (Practice parameter 2000) and all athletes completed a Post-Concussive Symptom Checklist (Aubry et al., (36))	**Normal controls** *n* = 8 Age = 22.1 ± 1.6	**Concussed group** *n* = 8 Age = 23.5 ± 3.38	—	No (Due to different units of presented result)
			**Results presented in percent of errors of individual participants**			
			—	Percent of errors from 2 concussed subjects were 2 SD above those of the non-concussed group (subject 6: RE = 43% and LE=17%; subject 8: RE = 13%).	—	
**Valadbeigi et al**. **(****33****)**	Yes Total *n* = 52	Participants in disease groups were diagnosed relapsing-remitting MS, by neurologists and MRI, with normal hearing threshold.	**Matched normal controls** *n* = 26 Age = 27.7 ± 5.2 M:F = N/A	**MS** *n* = 26 Age = 28.9 ± 4.1 M:F = N/A	—	No (Due to no numeric data provided)
			**Results were presented in msec, illustrated box plot without numeric data. Authors reported a significant difference between MS and control groups**.			

*GIN, gaps-in-noise; M:F, male:female; MS, multiple sclerosis; MTS, mesial temporal sclerosis; TLE, temporal lobe epilepsy; RE, right ear; LE, left ear; DurS, duration with small perceptual distance; DurL, duration with large perceptual distance; RTLE, right temporal lobe epilepsy; LTLE, left temporal lobe epilepsy. Age was presented in years; mean ± SD*.

### Temporal Ordering Tests

#### Duration Pattern Test

**Figure 3:**
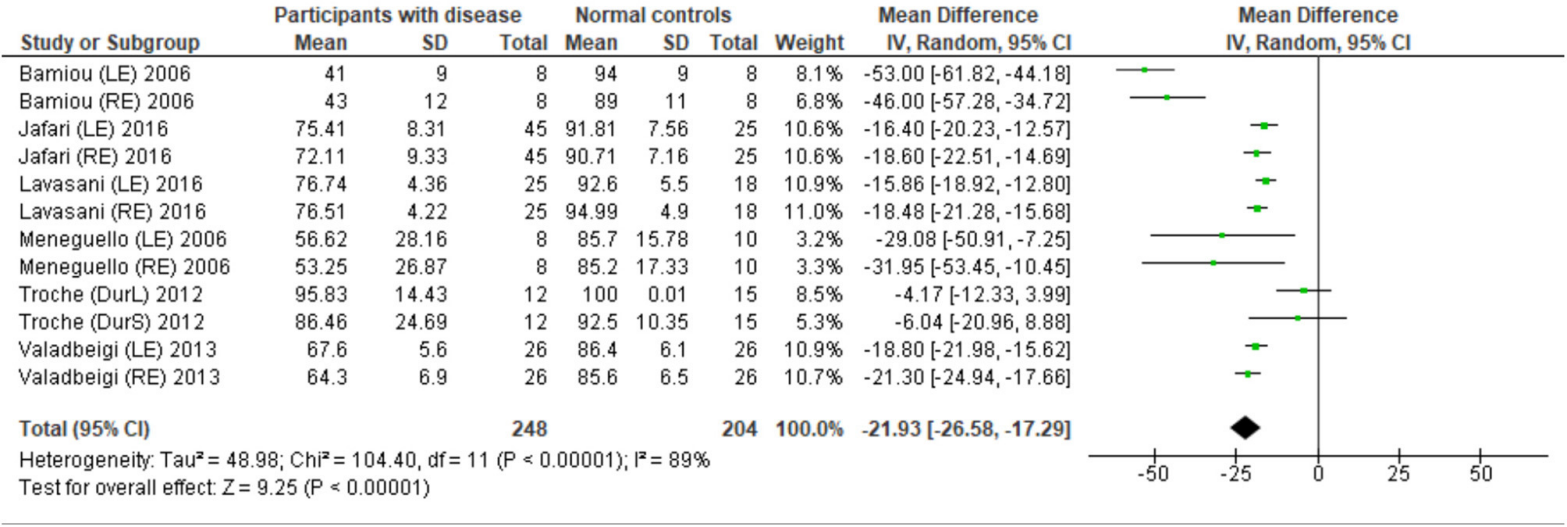
Forest plot of meta-analysis for duration pattern test (DPT), comparing between participants with documented brain disease and normal controls.

**Figure 4:**
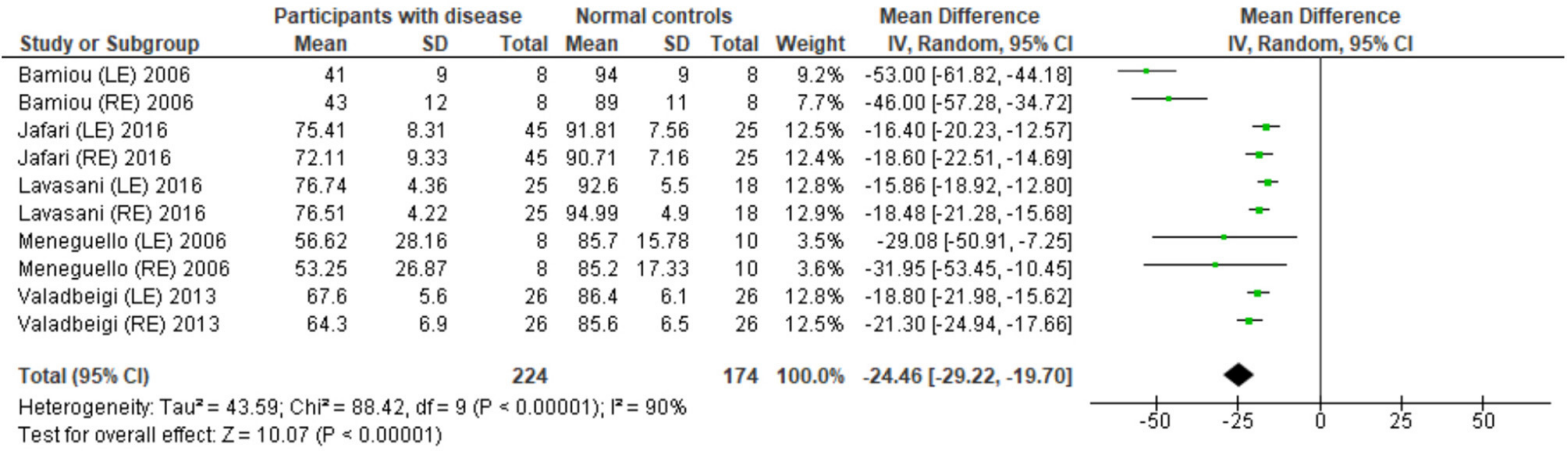
Forest plot of duration pattern test (DPT) for sensitivity analysis after excluding the studies from Troche et al. (31).

Eight studies (23, 25, 27, 28, 31–34) used DPT on participants with documented structural/functional brain disease (Table 2). Six of these studies (23, 25, 27, 28, 31, 33), which presented the result in the percent correct DPT and had control comparison, were further statistically analyzed (Figure 3). Jafari et al. (25) and Lavasani et al. (27) reported the DPT by side of brain pathology in terms of right brain damage (RBD) vs. left (LBD) and right temporal lobe epilepsy (RTLE) vs. left (LTLE), respectively. In order to conduct the meta-analysis, the results from both sides of brain pathology were combined. The DPT results from the studies of Jafari et al. (25), Lavasani et al. (27), Valadbeigi et al. (33), Bamiou et al. (23), and Meneguello, Leonhardt, and Pereira (28) were presented in separate ears, while Troche et al. (31) showed the overall result from both ears in the different test strategies, which were the duration with large (DurL) and small (DurS) perceptual distance, determined by the difference in pairs of tones by a large amount [i.e., 2,000 milliseconds (msec)] and a small amount (i.e., 500 msec), respectively. The mean performance of DPT in participants with diseases was significantly poorer than the controls with the mean difference of −21.93 (95% CI, −26.58 to −17.29). However, there was a high heterogeneity according to an *I*^2^ value of 89%. Afterward, all papers were reevaluated, and the cause of heterogeneity was suspected to arise from the study by Troche et al. (31) due to the different test strategies the authors used. A sensitivity analysis was thus conducted. However, the *I*^2^ was still high without significant change of overall effect (Figure 4).

#### Frequency Pattern Test

**Figure 5:**
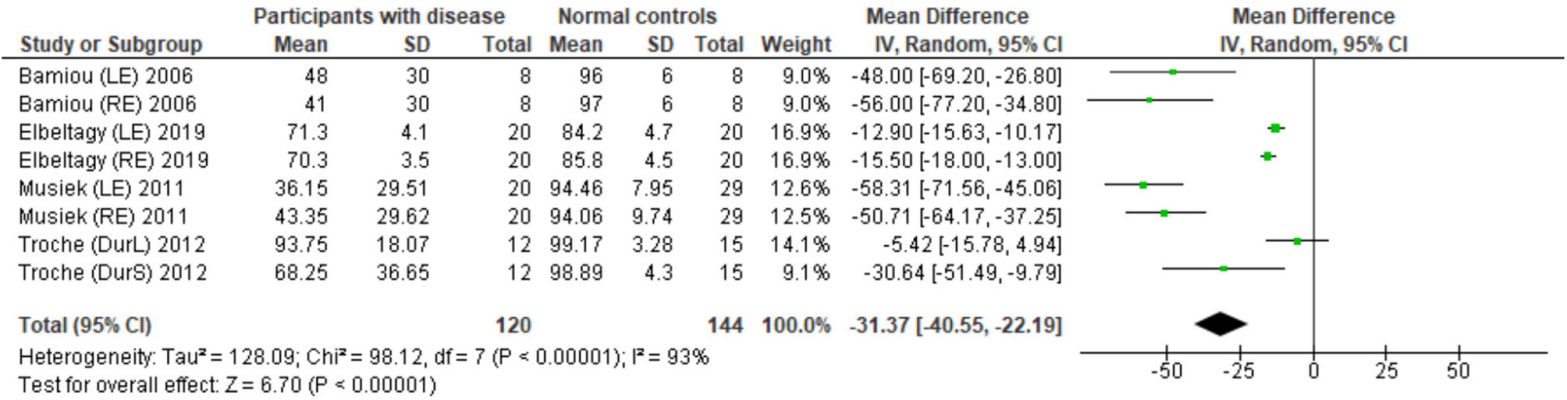
Forest plot of meta-analysis for frequency pattern test (FPT), comparing between participants with documented brain disease and normal controls.

**Figure 6:**
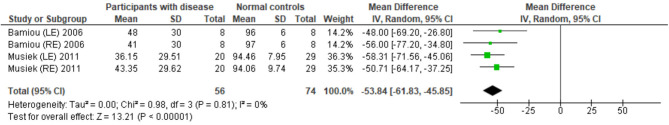
Forest plot of frequency pattern test (FPT) for sensitivity analysis after excluding the studies by Troche et al. (31) and Elbeltagy et al. (24).

The data extraction for FPT in participants with organic brain disease from seven studies (13, 23, 24, 29, 31, 32, 34) was presented in Table 3. Four studies (13, 23, 24, 31) that presented the results of FPT in percent correct were eligible for statistical analysis. The studies by Elbeltagyet al. (24), Musiek et al. (13), and Bamiou et al. (23) presented the results of FPT from each ear, while the study from Troche et al. (31) presented results from different test strategies. The forest plot of the meta-analysis for FPT is depicted in Figure 5. The control groups performed significantly better in FPT, with the mean difference of −31.37 (95% CI from −40.55 to −22.19). However, the heterogeneity was high, *I*^2^ = 93%. After all studies were reassessed, the study by Troche et al. (31) was suspected of causing heterogeneity due to the different strategies of testing. Also, the study by Elbeltagy, Gad, and Ismail (24) performed FPT in multiple sclerosis (MS) patients, which were different from the participants in the studies by Musiek et al. (13) and Bamiou et al. (23). Therefore, to perform the sensitivity analysis, the studies by Troche et al. (31) and Elbeltagy, Gad, and Ismail (24) were excluded. The results showed a significant reduction of heterogeneity from 93% to 0%, while the mean difference was still significantly better in the control group (mean difference = −53.84 with 95% CI of −61.83 to −45.85) (Figure 6).

### Temporal Resolution Test

**Figure 7:**
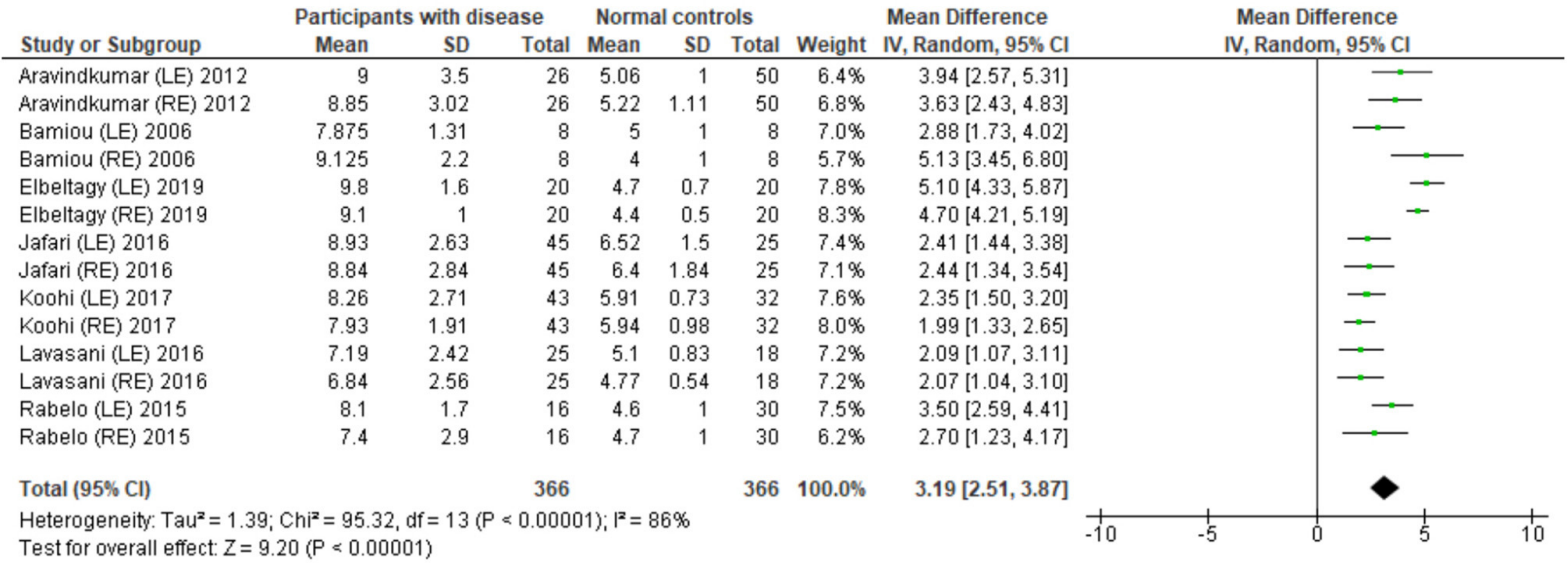
Forest plot of meta-analysis for gaps-in-noise (GIN), comparing between participants with documented brain disease and normal controls.

**Figure 8:**
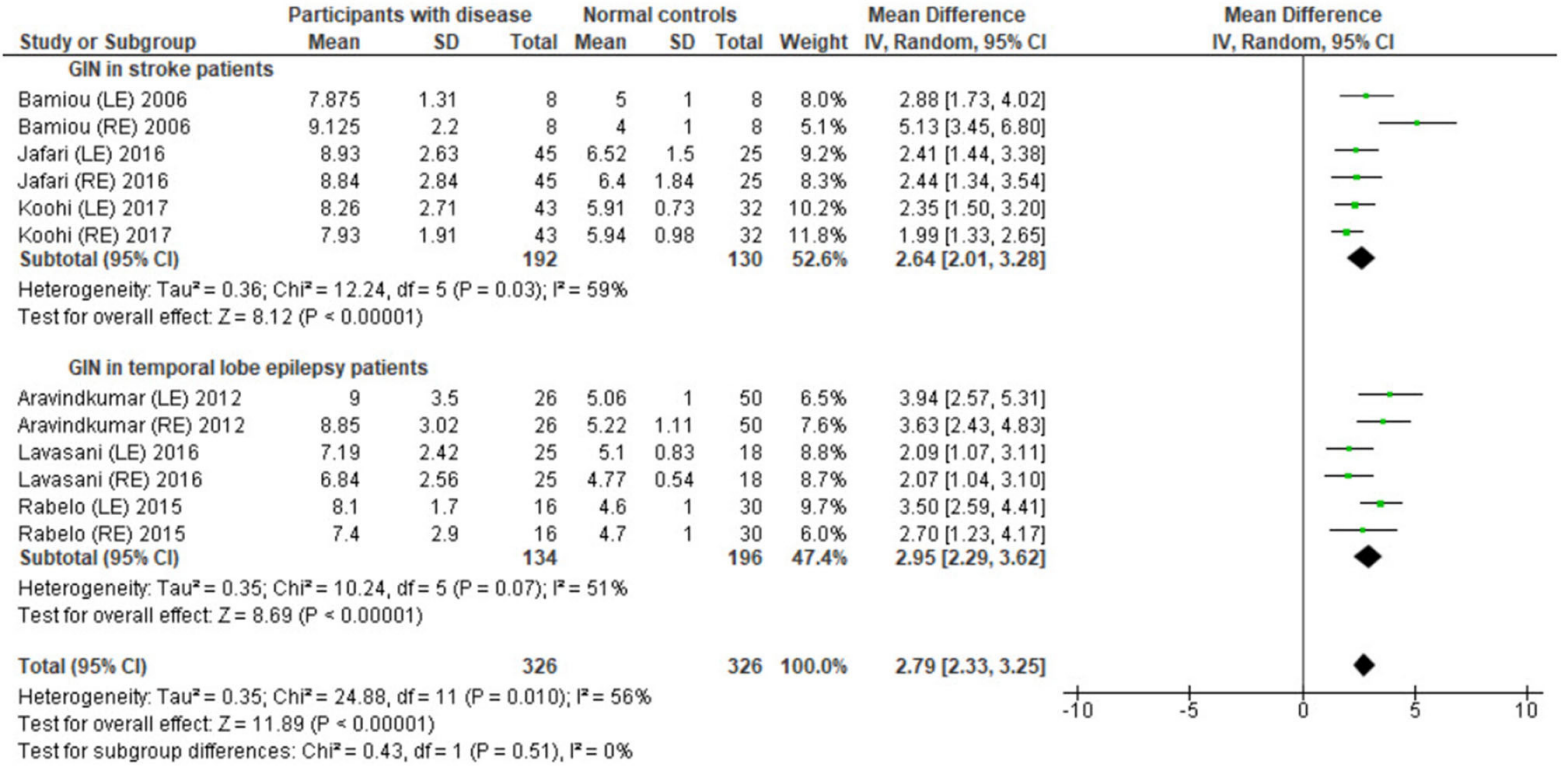
Forest plot of meta-analysis for gaps-in-noise (GIN) with subgroup analysis, categorized by the underlying conditions (stroke and temporal lobe epilepsy).

Several studies used the GIN test to assess temporal resolution processing in participants with documented structural/functional brain disease. The data extraction is shown in Table 4. Ten papers (12, 22–27, 30, 32, 33) were included, but only seven papers that presented GIN results in terms of thresholds (duration of the gap in milliseconds) were further processed in the meta-analysis. The studies by Rabelo, Weihing, and Schochat (30) reported the results separated by ears. Jafari et al. (25), Lavasani et al. (27), and Bamiou et al. (23) reported the GIN results separated by ears and sides of lesions. Data from both sides of lesions were combined together with the raw data from Koohi et al. (26) in order to conduct the meta-analysis. The forest plot from the meta-analysis showed better performance of GIN in the normal control groups with a mean difference of 3.19 msec (95% CI, 2.51 to 3.87) with high heterogeneity (*I*^2^ = 86%) (Figure 7). Due to the high heterogeneity, the subgroup analysis was done by categorizing into the stroke subgroup and TLE (or mesial temporal sclerosis) subgroup. The study by Elbeltagy, Gad, and Ismail (24), which reported the GIN result in MS patients, could not be included to these two groups. The heterogeneity decreased from the *I*^2^ of 86% to 59% in the stroke subgroup and 51% in the TLE subgroup (Figure 8). However, there was no significant subgroup effect (*p* = 0.51, *I*^2^ = 0%).

## Discussion

This study aimed to determine the efficacy of auditory temporal processing tests for detecting central auditory processing abnormalities in adults who have well-established and/or documented brain pathology. Confirmed pathology in the brain was used as the gold reference standard to determine the efficacy of the temporal processing tests in detecting auditory processing deficits. The results from three meta-analyses from DPT, FPT, and GIN tests all showed poorer central auditory processing abilities in individuals with documented brain pathology compared to normal controls. Overall, the meta-analysis provided essential evidence that DPT, FPT, and GIN are valid and sensitive clinical measures for central auditory assessment in adults. However, the meta-analysis could not be done in all included studies due to the limitation of different patterns of result presentation and groups of participants in studies. The discussion below shows more detail by topic.

### Temporal Ordering Tests

#### Duration Pattern Test Outcomes

The meta-analysis results in six studies (Figure 3) showed statistically poorer performance in participants with brain disorder. This result is in line with the report by Vermiglio (14) that analyzed the data from the study by Musiek et al. (7) and reported 85.7% sensitivity and 92.0% specificity of DPT using the participants with documented auditory cortex involvement as a gold standard. However, this meta-analysis also showed the presence of a high heterogeneity of data possibly due to the heterogeneity of the neurological brain disorders included.

Interestingly, Troche et al. (31) performed different DPT strategies using DurS and DurL. Although there was no statistical difference between groups, subjects with Parkinson's disease performed better in DurL compared to DurS. The basal ganglia may contribute to auditory rhythm detection (37); however, Parkinson's disease can also cause cognitive decline (38). While the meta-analysis provided some evidence for the potential of DPT for diagnosing CAPD, clinicians should be aware that the DPT results could be affected by the different DPT strategies of testing the characteristics of brain pathology and the involved locations of the underlying brain diseases.

#### Frequency Pattern Test Outcomes

According to the meta-analysis and sensitivity analysis (Figures 5, 6), the study by Troche et al. (31) and Elbeltagy et al. (24) were the cause of heterogeneity. The study by Troche et al. (31) differed from the other two studies (13, 23) in terms of the characteristics of the underlying brain pathology (Parkinson's disease) and test strategies, while the study by Elbeltagy et al. (24) performed FPT in participants with MS. Bamiou et al. (23) and Musiek and Chermak (13) conducted FPT in participants with confirmed brain lesions that involved the auditory region. According to the meta-analysis results, these participants performed significantly worse in FPT than the controls with 0% of heterogeneity. Previous studies (13, 14) showed 75.0% sensitivity and 100.0% specificity of FPT using documented auditory area involvement as a gold standard. Therefore, the present results strongly indicate that FPT is directly related to the function of the auditory cortex, rather than other cerebral regions.

### Temporal Discrimination Tests

Using participants with confirmed brainstem or cerebral lesion as a gold standard, Musiek et al. (12) reported 67% of sensitivity and 94% of specificity for the GIN test and also suggested that the GIN test was more sensitive to cortical lesions (12). A recent meta-analysis of GIN test in participants with neurological conditions also reported good sensitivity and specificity in detecting pathology in the CANS (39). The meta-analysis from seven studies in our review also supports the high specificity of GIN, showing significantly poorer GIN results in the disease group. We conducted a subgroup analysis by categorizing studies into the stroke subgroup and the TLE subgroup that decreased heterogeneity in both subgroups. We propose that different characteristics of brain diseases may differentially affect the GIN results. Furthermore, the observed heterogeneity could arise due to the diversity in normative GIN threshold, difference of technical procedures, and subject training (6). The meta-analysis by Filippini et al. (39) similarly showed different efficiency of the GIN test in detecting abnormality in the CANS in different underlying neurological conditions, with a higher efficiency for epilepsy, followed by stroke and blast exposure (39).

### Strengths, Limitations, and Suggestions for Further Studies

The results of the meta-analysis showed high sensitivity of DPT, FPT, and GIN test in detecting abnormalities in auditory processing in participants with brain pathology. Of interest, a recent study (40) identified significant heritability of 0.72 for another measure of temporal resolution, backward masking. These temporal processing tests may hold promise as potential biomarkers of central auditory function. They may also reliably identify the site of lesion and/or level of central auditory processing deficits in adults with neurological conditions. The study limitations included a great variety of testing strategies and differences in units of the reported results. For example, DPT has been reported in mean percent correct or the percentage of participants with abnormal results. In addition, the normative value references and cut points also differed among studies, providing difficulties in data extraction. A standard pattern of result reporting is required to facilitate a meta-analysis. The test strategies should be standardized with international protocols in order to reduce factors that may interfere with the test results. Although there were variations in units of reporting results, each meta-analysis included only studies with similar reporting units.

The various underlying brain conditions are another possible cause for high heterogeneity. This meta-analysis included studies with a variety of brain pathologies, including stroke, MS, Parkinson's disease, and TLE. Although the results demonstrated that brain pathology caused lower performance in temporal processing, there was high heterogeneity of recruited studies for meta-analysis in DPT, FPT, and GIN. This could indicate that different brain conditions may differentially affect performance in temporal processing tests. Also, there was lack of information about the medications and treatments for individuals in several studies. Stroke and mass lesions may lead to focal CANS lesions that directly affect auditory temporal processing. MS is a progressive disease causing demyelination and axonal scarring (41) that could preferentially affect myelin-rich regions, including corpus callosum, medial longitudinal fasciculus, and periventricular regions (42). The timing in CANS could thus be affected by MS, and various degrees of severity in auditory temporal processing could be observed in MS patients, depending on the number of involved areas and severity of MS pathology along CANS. Parkinson's disease is a progressive neurodegenerative disease characterized by the degeneration of the nigrostriatal dopaminergic system (43). Basal ganglia may affect the temporal auditory processing for complex rhythm (37). The review by De Groote et al. (44) also showed impaired temporal processing in patients with Parkinson's disease. TLE is characterized by seizure in the temporal lobe caused by an imbalance between the excitatory and inhibitory (41, 42). Seizure negatively affects the temporal lobe regions and later causes their degeneration and sclerosis (42). Furthermore, a variation of neuronal activity synchronization is also found, resulting in cognitive function decline (41), which may also impact on the temporal processing ability. Progression of MS and Parkinson's disease or uncontrolled TLE also progressively degrades the auditory temporal processing function.

Categorizing the underlying brain diseases for subgroup analysis may reduce heterogeneity and clarify the effect of particular brain conditions on the temporal processing. Furthermore, the side of brain lesions may affect the temporal processing differently. However, most studies reported the overall results of temporal processing without considering the laterality of lesions. Even though the studies by Jafari et al. (25), Lavasani et al. (27), Aravindkumar et al. (22), and Bamiou et al. (23) reported the results regarding the location of the lesions, the results were combined in order to proceed with the meta-analysis. For example, Lavasani et al. (27) reported significantly worse DPT performance in the LTLE group compared to RTLE participants, but there was no significant difference of DPT between RTLE and controls. However, this study reported no significant difference of GIN in LTLE and RTLE groups. In contrast, the study by Aravindkumar et al. (22) reported a significant difference of GIN among left MTS, right MTS, and control groups. Therefore, the effect of lateralization on temporal processing should be further explored by using a meta-analysis.

According to our search results, there were only a few studies that directly compared the temporal processing performance between disease groups and controls. Several studies aimed to assess the efficacy of different testing strategies (such as efficacy of using different types of stimuli) rather than evaluate the central auditory function and these studies were excluded from this present study. Other papers were excluded, as they were judged to be of poor quality and/or subject to bias due to limitations in study design. Overall, there is a need for additional high-quality evidence (i.e., from randomized controlled trials using standardized outcome measures for CAPD) to demonstrate the effectiveness of CAPD diagnostic tests. Such evidence is pivotal to guide service delivery models for evidence-based clinical practice.

## Conclusion

According to our meta-analysis results, the DPT, FPT, and GIN are sensitive detectors of auditory processing deficits in individuals with brain pathology. Different types of brain pathology and different sites of lesion may differentially affect these test results, as this review also provided strong evidence that FPT is sensitive to the function of the auditory cortex, rather than other cerebral regions. By extrapolation, these three sensitive clinical measures may have the potential to detect temporal resolution and temporal ordering deficits, indicating CANS abnormalities, in adult individuals without obvious brain lesions documented on imaging, and this should be further investigated.

Clinicians should be cautioned to interpret these test results in the context of other patient characteristics (e.g., cognition) and be aware that not all brain pathologies will lead to deficits in auditory processing function, depending on its location characteristics and natural history of the neurological disorder.

## Data Availability Statement

The raw data supporting the conclusions of this article will be made available by the authors, without undue reservation.

## Author Contributions

SC conceptualized and designed the study, searched the databases, collected and organized the data, assessed the quality of studies, performed the statistical analysis and systematic review, drafted the initial manuscript, and revised the manuscript. D-EB coordinated and supervised data collection, critically reviewed, and revised the manuscript. NK conceptualized and designed the study, searched the databases, organized the data, assessed the quality of studies, and critically revised the manuscript. All authors read and approved the final manuscript.

## Conflict of Interest

The authors declare that the research was conducted in the absence of any commercial or financial relationships that could be construed as a potential conflict of interest.
